# Whole Genome Sequencing of Drug-Resistant *Vibrio cholerae* Serotype Ogawa from an Outbreak in Khyber Pakhtunkhwa

**DOI:** 10.3390/pathogens15010039

**Published:** 2025-12-29

**Authors:** Aftab Ali, Momin Khan, Taj Ali Khan, Sajjad Ahmad, Noor Rahman, Aiman Waheed, Taane G. Clark

**Affiliations:** 1 Department of Microbiology, Institute of Pathology and Diagnostic Medicine, Khyber Medical University, Peshawar 25100, Pakistan; aftaba027@gmail.com (A.A.);; 2 Center of Biotechnology and Microbiology, University of Peshawar, Peshawar 25120, Pakistan; 3 Faculty of Infectious and Tropical Diseases, London School of Hygiene & Tropical, London WC1E 7HT, UK

**Keywords:** *Vibrio cholerae*, diarrhoea, MDR, genomic epidemiology

## Abstract

Background: Cholera, caused by *Vibrio cholerae*, remains endemic in many developing countries, including Pakistan. The extensive use of antibiotics has led to the emergence of antimicrobial resistance in *V. cholerae*, limiting available treatment options. In this study, we performed molecular characterisation of antibiotic-resistant *V. cholerae* serotype Ogawa isolates from a recent cholera outbreak in Khyber Pakhtunkhwa, Pakistan. Methodology: Suspected cholera stool samples were collected from hospitalised patients at various district hospitals of Khyber Pakhtunkhwa Province (KPK), Pakistan. The samples were transported to the Public Health Reference Microbiology Laboratory at Khyber Medical University, Peshawar. *V. cholerae* were identified based on colonial morphology, Gram staining, and biochemical tests using EPI 10E. For serotype identification, monovalent antisera were used. Antibiotic susceptibility testing (AST) was performed using CLSI M45 and EUCAST guidelines. DNA was extracted from pure colonies of multidrug-resistant (MDR) *V. cholerae* and subjected to whole-genome sequencing (WGS) for genomic characterisation using an Illumina MiSeq platform. Results: Of the 350 active diarrheal cases investigated, 70 were confirmed as *V. cholerae*. The outbreak was initially reported in Dir and was subsequently followed by a high incidence of cholera in the Peshawar district of KPK. All strains belong to the Ogawa serotype, which shows high antibiotic resistance, particularly to ampicillin (*n* = 62, 88.57%), Sulfamethoxazole/Trimethoprim (*n* = 60, 85.71%), Erythromycin (*n* = 59, 84.29%), and Tetracycline (*n* = 53, 75.71%). The lowest resistance was against Meropenem (*n* = 1, 1.4%), followed by amikacin (*n* = 7, 10.0%) and levofloxacin (*n* = 13, 18.57%). Furthermore, 34 (48.57%) of the isolates were MDR, while 13 (18.57%) were extensively drug-resistant. Six samples were selected for whole-genome sequencing. The selection of six *V. cholerae* samples for WGS was based on their drug resistance pattern and origin of isolation. At the genomic level, all sequenced *V. cholerae* strains harboured multiple antimicrobial resistance determinants. Quinolone resistance was associated with mutations and genes in *gyrA*, *gyrB*, *parC*, and *parE*; resistance to sulfamethoxazole–trimethoprim with *folA*, *folP*, and *dfr;* tetracycline resistance with tetA and tet35; chloramphenicol resistance with *catB* and *S10p*; and aminoglycoside resistance with *hns*, *S12p*, and *gigB*. In addition, β-lactam resistance was linked to the presence of efflux and β-lactamase genes, including *blaSHV* and *mox-3*. Mutations were identified in *gyrA* at positions S83I, S177A, and S202A, and in *parC* at positions S85L and I231V. Collectively, the presence of these resistance determinants likely enables *V. cholerae* to survive exposure to high concentrations of multiple antibiotics. Conclusions: Our *V. cholerae* isolates showed close genetic relatedness to previously sequenced strains from Pakistan (2010 and 2022), as well as to recently reported international strains from the USA, Australia, and China. These findings highlight both the long-term persistence of these lineages within Pakistan and their international dissemination, likely facilitated by globalisation.

## 1. Introduction

Cholera is an acute watery diarrheal disease caused by a Gram-negative bacterium, *V. cholerae*, associated with severe morbidity and mortality [1]. It is responsible for more than 3 million cases globally each year, with annual mortality estimated at approximately 100,000 deaths. A twofold increase in incidence has been reported in several African countries, largely driven by inadequate sanitation systems and limited access to safe drinking water across affected communities [2,3]. Given the severity of cholera, the World Health Organization (WHO) recommends oral rehydration therapy for mild to moderate cases and intravenous rehydration for severe cases [4]. The *V. cholerae* pathogen is mostly transmitted from person to person by faecal oral contamination or the consumption of contaminated food, while transmission from the environment to a person is mainly due to water reservoir contamination [5]. Contributing factors to the transmission of cholera are extreme environmental events (floods, earthquakes) [6,7], poor sanitation systems, a lack of pure drinking water, humanitarian crises, health emergencies, and overloaded health systems [8]. The severity of cholera depends on the pathogen, serotypes, virulence factors, infection dose, and host immunity. Based on the cell surface lipopolysaccharide antigen (O antigen), *V. cholerae* has been differentiated into more than 200 serogroups [7]. However, serogroups O1 and O139 are most commonly associated with cholera outbreaks and epidemics in endemic regions [9]. The O1 serogroup comprises two biotypes: Classical and El Tor. The sixth cholera pandemic was caused by the Classical biotype, whereas the ongoing seventh pandemic is attributed to the El Tor biotype. El Tor is a modern variant that carries virulence genes, including the cholera toxin gene originally found in the Classical biotype [9]. Both the Classical and El Tor biotypes are further divided into the Inaba and Ogawa serotypes, based on differences in their O-antigen structure.

While the history of modern cholera dates back to 1817, descriptions of cholera-like illnesses appear as early as the time of Hippocrates, around 300–500 BC [10]. The O1 serogroup caused the earlier outbreaks and is associated with all seven cholera pandemics since 1817 [9]. In contrast, the first cases of *V. cholerae* O139 were reported in India in 1990 and in Pakistan in 1992. However, most recent cholera outbreaks in both Pakistan and India continue to be dominated by O1 serotypes, with O139 detected only rarely [11,12,13,14]. Genomic studies have identified two new clades of *V. cholerae* circulating in Pakistan (PCI and PCII), which can be distinguished by specific mobile genetic elements, insertion sequences, toxin-encoding loci, SXT elements, and genomic deletions that influence both pathogenicity and antimicrobial resistance (AMR) profiles [15]. In 2022–2023, Malawi experienced one of its most severe cholera outbreaks. Genomic epidemiology showed a close relationship between the Malawi isolates and strains sequenced in Pakistan in 2022, as well as two Australian cases linked to recent travel to Pakistan. Malawi isolates shared similar serotypes, sequence types, AMR patterns (phenotypic and genomic), and SNP-based phylogenetic profiles with strains from Pakistan [16,17]. Recent outbreaks in neighbouring Bangladesh have also shown strong genomic relatedness to strains from Pakistan, including a reported serotype shift from Inaba to Ogawa [18]. Yemen likewise experienced a major cholera outbreak in 2022–2023; while its AMR profile resembled that of Pakistani isolates, genomic and epidemiological evidence suggests an origin linked to Bangladeshi and Indian lineages [19].

Pakistan, a developing country, faces a high burden of AMR driven by the widespread misuse of antibiotics in both healthcare and poultry sectors, compounded by inadequate quality-control measures at practitioner and governmental levels. Over the past decade, multidrug-resistant (MDR) and extensively drug-resistant (XDR) bacterial strains have been increasingly reported across the country, posing a significant threat to global health [20].

The Khyber Pakhtunkhwa (KPK) province of Pakistan has been severely affected by multiple natural disasters. The 2005 earthquake (21), followed by the extensive flooding in 2010, significantly compromised living conditions and public health standards, rendering the population increasingly vulnerable to infectious diseases, including cholera. In addition to natural disasters, the region has endured prolonged periods of armed conflict, which resulted in mass displacement and large-scale population movements [21]. Poor sanitation, overcrowded settlements, and overburdened healthcare facilities have contributed to a rise in gastrointestinal infections, with cholera becoming a particular concern. Since the first genomically characterised cholera case reported in 2010, the disease has become endemic in KPK; however, it has received limited attention at policy, surveillance, and research levels.

This study aims to characterise the AMR profiles and genomic relatedness of *V. cholerae* Ogawa strains isolated during the 2023 cholera outbreak in KPK, Pakistan.

## 2. Materials and Methods

### 2.1. Sampling

To determine the prevalence of *V. cholerae* in KPK, stool samples from suspected cholera cases were collected from all districts using a convenient sampling approach, focusing on hospitalised patients. Before sample collection, approval was obtained from the ethical review board of the IPDM-KMU (No. KMU/IPDM/ICE/2022-11). The study included patients presenting with acute watery diarrhoea within the designated outbreak area. Patients with drug-induced diarrhoea, dysentery, hemorrhagic diarrhoea, or those residing outside the defined study population were excluded. Patients were informed about the sampling procedure and the purpose of the study, after which written consent was obtained from the patient or their attendant. Stool samples were then collected in leak-proof containers using appropriate sanitation practices and standard microbiological protocols [22]. The collected samples were transported to PHRL-KMU in Cary Blair medium for laboratory analysis. Strain identification was performed based on growth characteristics on TCBS agar, Gram staining, biochemical tests, and the API E10 kit (BioMérieux, Marcy-l’Étoile, France). Serotyping was conducted using Ogawa, Inaba, and Bengal antisera supplied by Denka Seiken Co., Ltd., Tokyo, Japan.

### 2.2. Antibiotic Sensitivity Testing to Determine the Susceptibility of V. cholerae

Antibiotic sensitivity testing was performed by the Disc Diffusion method on Müller–Hinton agar (Oxoid, UK) [23]. A 0.5 McFarland turbidity standard was prepared, and culture suspensions were adjusted accordingly before being inoculated onto MHA plates using sterile cotton swabs. Antibiotic discs were then applied, and plates were incubated for 24 h. The antibiotics tested (µg/disc) included ampicillin (10), Cefixime (10), Cefotaxime (30), sulfamethoxazole–trimethoprim (30), tetracycline (30), chloramphenicol (30), Colistin (30), ciprofloxacin (30), levofloxacin (5), and amikacin (30). All discs were obtained from Oxoid™ (Thermo Fisher Scientific, Loughborough, UK). Antimicrobial susceptibility of each isolate was interpreted as sensitive, intermediate, or resistant according to CLSI M45 guidelines and, where available, EUCAST breakpoints (v15.0) [24,25,26,27]. For colistin, disc diffusion results were reported descriptively only, as CLSI and EUCAST do not recommend disc diffusion-based interpretive categorisation for *Vibrio* spp. Similarly, cefixime results were evaluated descriptively due to the limited organism-specific interpretive criteria. MDR and XDR classifications for *V. cholerae* were assigned according to the European Centre for Disease Prevention and Control (ECDC) guidelines [28].

### 2.3. Genomic DNA Extraction

**Figure 1:**
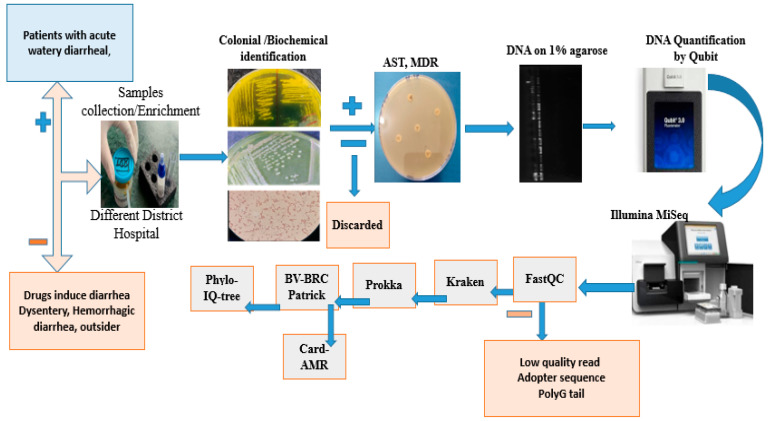
Sample selection and processing flowchart.

Genomic DNA of *V. cholerae* was extracted from overnight cultures grown at 37 °C in Luria broth. Following centrifugation, the supernatant was discarded, and DNA was isolated from the cell pellet using the QIAamp DNA extraction kit (Qiagen, Germany). DNA quality was assessed on a 1% agarose gel, and quantification was performed using a Qubit fluorometer with the dsDNA HS assay kit (Thermo Scientific, Waltham, MA, USA). Purified DNA samples were then submitted to the China Institute of Medical Science (CIMS) for whole-genome sequencing using an Illumina MiSeq platform (Figure 1).

### 2.4. Genome Analysis of NGS Data

Raw sequencing reads were assessed for quality using FastQC (v0. 12.1) and MultiQC (v. 1.19) following the standard protocols [29]. Adapter sequences and low-quality bases were removed using Trimmomatic (v0.36) with a sliding-window cutoff of 4:20, minimum read length of 50 bp, and leading/trailing base quality threshold of 20. High-quality filtered reads were assembled de novo using SPAdes (v3.14.0) with default parameters, after which contigs with <10-fold coverage and <500 bp length were excluded [30]. Genome completeness and contamination were assessed using CheckM2 software [31], and assembly quality statistics were generated using the QUAST tool (v3.9). Species identification was performed using Kraken2 (v2.1.2) against the standard database, employing a minimum confidence score of 0.1 and a ≥90% identity threshold for taxonomic assignment. Genome annotation was conducted with Prokka (v1.14.6) under default bacterial settings [32]. AMR genes were identified using PATRIC, with detection thresholds of ≥80% coverage and ≥90% sequence identity. For WGS-based phylogeny, in-house *V. cholerae* isolates were uploaded and annotated in BV-BRC tools. A codon-based phylogenomic tree was generated using the Bacterial Codon Tree pipeline, incorporating (1) our in-house isolates and (2) publicly available *V. cholerae* genomes from 2020 to 2024, with default parameters (MLST-based marker selection, 1000 bootstrap replicates, and an ANI ≥ 95% inclusion threshold) [33]. The resulting Newick-format tree was visualised and annotated in iTOL (v7) [34] (Figure 1).

## 3. Results

### 3.1. Identification of Cholera Outbreak in Khyber Pakhtunkhwa, Pakistan

**Figure 2:**
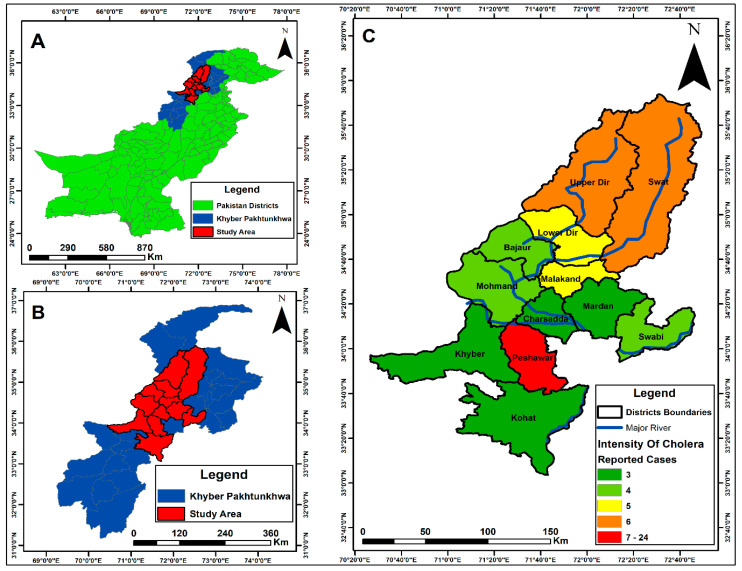
The map was created using the Pakistan district shapefile in ArcGIS (v10.5). (**A**) shows the map of Pakistan; (**B**) displays Khyber Pakhtunkhwa (KPK) province; (**C**) highlights the study area within KPK. Encircled areas indicate the districts from which *V. cholerae* samples were received, while the colour intensity represents the number of confirmed cases in each district. Blue lines depict rivers, with one originating from Upper Dir and the other from the Swat region.

KPK is a disaster-prone province of Pakistan and remains highly vulnerable to gastrointestinal infections, particularly cholera, which has become endemic in the region. Between April 2023 and November 2023, a total of 350 stool samples from suspected cholera cases were received at the PHRL. Samples were collected from hospitalised patients of all ages and both genders across multiple district hospitals in KPK. Of the 350 samples, 70 were confirmed as *V. cholerae* using a rapid immunochromatographic test (ICT), followed by biochemical identification based on colonial morphology on TCBS agar, Gram staining, and confirmation using the API 10E kit. Serotyping of these biochemically confirmed isolates revealed that all belonged to the Ogawa serotype. Most confirmed cases were from the Peshawar district—the province’s largest city, which experiences substantial population movement from within the country and internationally due to its airport. Other affected districts included Swat, Dir, Mardan, and Charsadda, with additional locations shown on the map (Figure 2).

The outbreak was initially reported in Dir, when PHRL-KMU received the first confirmed *V. cholerae* case from the District Headquarters Hospital in Timergara. Subsequent cases were reported from Swat, Malakand, and other districts. However, the highest number of cases occurred in Peshawar, likely due to the direction of water flow and the large-scale movement of people to the city for various activities

### 3.2. Antibiotic Resistance and Sensitivity Profile of V. cholerae Isolates

**Figure 3:**
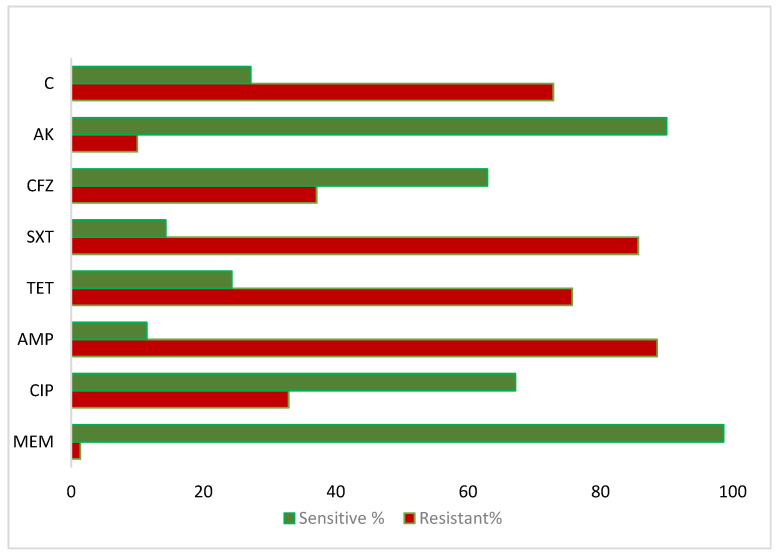
The *V. cholerae* antibiotics sensitivity profile against the antibiotics: C-Chloramphenicol, AK-Amikacin, CFZ-Cefotaxime, SXT-Sulfamethoxazole-Trimethoprim, TET-Tetracycline, AMP-Ampicillin, CIP-Ciprofloxacin, and MEM-Meropenem.

The antibiotic susceptibility profile of the isolated *V. cholerae* strains, determined by the disc diffusion method, showed high resistance to ampicillin (62/70, 88.57%), sulfamethoxazole–trimethoprim (60/70, 85.71%), erythromycin (59/70, 84.29%), tetracycline (53/70, 75.71%), and chloramphenicol (51/70, 72.86%). The lowest resistance was observed against meropenem (1/70, 1.4%), followed by amikacin (7/70, 10.0%) and levofloxacin (13/70, 18.57%) (Figure 3). Among the resistant isolates, 34/70 (48.57%) displayed MDR, defined as resistance to three classes of antibiotics, while 13/70 (18.57%) exhibited XDR, being resistant to four or more antibiotic classes. Antibiotics lacking validated interpretive criteria (including Colistin and Cefixime) were excluded from MDR/XDR classification.

### 3.3. Comprehensive Antimicrobial Resistance Gene Profiling of V. cholerae via Whole-Genome Sequencing

**Figure 4:**
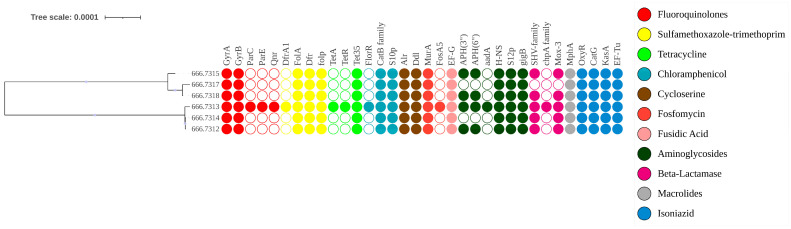
The AMR gene profile of *V. cholerae* is shown alongside the phylogeny of the study isolates generated on BV-BRC tools. Samples 666.7609 to 666.7318 represent the annotated isolates. The presence or absence of each AMR gene is indicated by filled or empty circles, respectively. Gene names are displayed above each circle, and the colour and intensity of the circles correspond to the class of antibiotics affected by the respective resistance genes.

Following WGS and annotation of the targeted six *V. cholerae* strains, AMR genes were identified using PATRIC via BV-BRC. Strain 666.7613 was found to be MDR, with some isolates exhibiting XDR, consistent both phenotypically and genotypically. Phenotypic resistance observed in AST was confirmed genomically through the presence of corresponding resistance genes. The AMR genes commonly detected across all strains included for: Fluoroquinolones (*gyrA*, *gyrB)*, Sulfonamides/Trimethoprim (*folA*, *folP*, *dfr*), Tetracycline (*tet35*), Chloramphenicol (*catB* family, *S10p*), Cycloserine (*alr*, *ddl*), Fosfomycin (*murA*), Aminoglycosides (*H-NS*, *S12p*, *gigB*), and Macrolides (*mphA*). Genes that were not phenotypically tested but were present in all isolates included isoniazid-related loci such as *oxyR*, *katG*, *kasA*, and *Ef-Tu* (Figure 4). Less frequently detected resistance genes included *parC*, *parE*, *tetA*, *tetR*, *aadA*, and the *CphA* family. Notable mutations associated with fluoroquinolone resistance were identified (*gyrA*: S83I, S177A, S202A; *parC*: S85L, I231V). This analysis highlights the concordance between phenotypic and genotypic resistance and provides a detailed profile of the resistance determinants in these *V. cholerae* isolates.

### 3.4. Phylogenomic Analysis of V. cholerae Reveals Global Evolutionary Linkages

**Figure 5:**
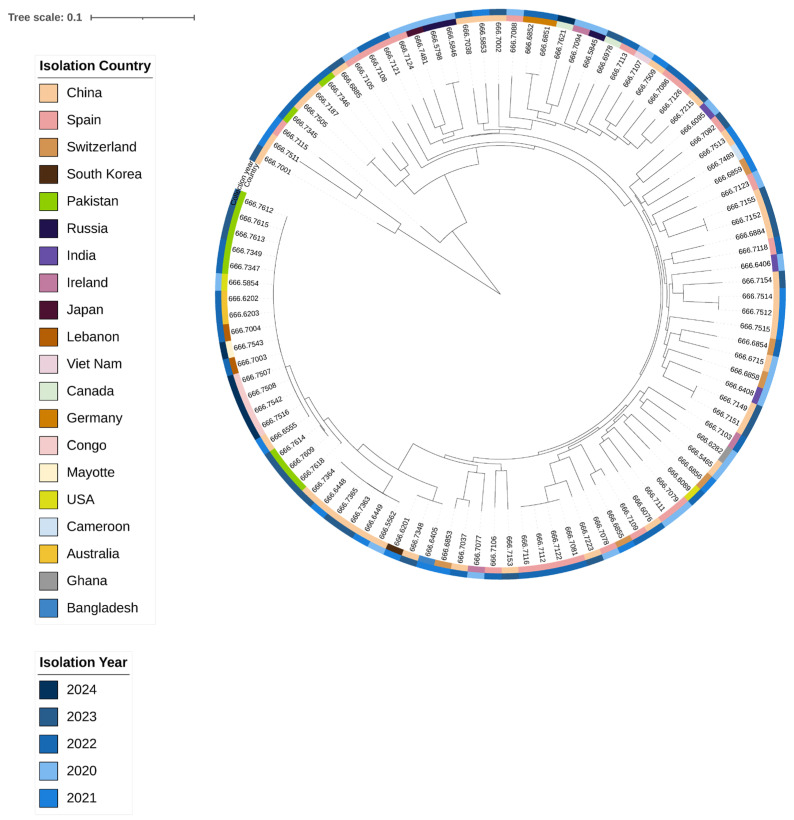
The maximum likelihood phylogenetic tree was constructed and annotated in iTOL. Sample origins are indicated on the left, while collection years are shown on the right. The tree in the centre illustrates that our isolates cluster tightly with Chinese strains, and this lineage is closely related to strains from the USA, Australia, Lebanon, and the Democratic Republic of Congo.

A maximum likelihood phylogenetic tree was constructed based on genome-wide SNPs. Sequences were mapped to the reference genome from Bangladesh (accession No. AE003852-3) and compared with *V. cholerae* genomes sequenced globally between 2020 and 2024. The consensus tree shows that all study isolates (666.7609, 666.7612, 666.7613, 666.7614, 666.7615, and 666.7318) cluster within the same clade, with minor divergence among their sub-branches. Strains 666.7609, 666.7614, and 666.7318 form a close cluster with a 2022 Chinese isolate, suggesting a recent common evolutionary ancestor and potential localised evolution and transmission between China and KPK, Pakistan. In contrast, genomes 666.7612, 666.7613, and 666.7615 are closely related to previously sequenced Pakistani strains (2022) as well as isolates from the USA (2020), China (2021), Australia (2022), and Lebanon (2022). This cluster has also recently spread to France (2024) and Congo (2024), with minimal divergence, indicating strong evolutionary linkage among these isolates (Figure 5).

## 4. Discussion

Cholera remains endemic in Pakistan, particularly in the northern regions, due to geography, frequent natural disasters, conflicts, and inadequate water and sanitation systems. Excessive and uncontrolled antibiotic use, compounded by systemic issues such as insufficient regulation, lack of culturally sensitive healthcare practices, inappropriate staffing, and high incentives from pharmaceutical companies, has contributed to the emergence of drug-resistant bacterial strains. Similarly, *V. cholerae*, once sensitive to first-line tetracycline, now shows high resistance to tetracycline and many other antibiotics, posing a significant public health threat. In 2023, KPK experienced severe flooding, followed by a cholera outbreak that initially emerged in the Dir district and subsequently spread across multiple districts, with the highest incidence reported in Peshawar.

In the current study, *V. cholerae* isolates exhibited high levels of antibiotic resistance: Ampicillin 88%, Sulfamethoxazole-Trimethoprim 85%, Erythromycin 84%, Tetracycline 75%, and Chloramphenicol 72%. The lowest resistance was observed against Meropenem (1.4%), Amikacin (10%), and Levofloxacin (18%). Although resistance to colistin and cefixime among clinical *V. cholerae* isolates ranged from 30–40% based on approximate zone diameter measurements, these findings are reported descriptively due to the absence or limitations of validated interpretive criteria for *V. cholerae* in CLSI and EUCAST guidelines. Comparable resistance patterns have been reported internationally. In Zimbabwe, MDR *V. cholerae* strains from the 2020 outbreak were largely resistant to tetracycline, ciprofloxacin, and even fourth-generation cephalosporins [35]. In Ethiopia, resistance was reported as Ampicillin 100%, SXT 60%, Tetracycline 50%, and Ciprofloxacin 17% [36]. In Kenya, resistance to Cotrimoxazole, Tetracycline, Ampicillin, and Erythromycin was 99%, 97%, 89%, and 53%, respectively, while isolates remained fully sensitive to Ciprofloxacin and Gentamicin, with high sensitivity to Ceftriaxone (97%) and Streptomycin (96%) [37]. In India, Ampicillin resistance ranged from 75 to 100%, with similar patterns for Tetracycline and Sulfamethoxazole-Trimethoprim (75%), while Ciprofloxacin and Gentamicin resistance remained below 25% [38].

In the current study, 34 (48.57%) *V. cholerae* isolates exhibited MDR, showing resistance to more than three antibiotic classes, predominantly Ampicillin, Tetracycline, and Sulfonamides. Additionally, 13 (18.57%) isolates displayed XDR, resistant to more than four antibiotic classes, including Ampicillin, Tetracycline, Sulfonamides, Fluoroquinolones, and Erythromycin. All isolates belonged to the Ogawa serotype. In Bangladesh, MDR *V. cholerae* accounted for 175 (28.1%) of isolates, with approximately 99% showing resistance to Ampicillin, Erythromycin, and Sulfonamides [39]. MDR strains have also been reported in recent outbreaks in Zimbabwe and Yemen, where the Yemeni strains carry plasmids and SXT elements harbouring MDR genes [19,35].

Genomic analysis of *V. cholerae* indicates that antibiotic resistance arises from either single or multiple mutations in target genes or the acquisition of AMR genes via mobile genetic elements such as plasmids, transposons, integrative conjugative elements, and integrons [40]. To assess AMR gene diversity, whole-genome sequences were annotated in BV-BRC, and AMR genes were identified using PATRIC software. Sample 666.7613 exhibited the widest array of AMR genes, consistent with an XDR phenotype, while the remaining five strains displayed MDR patterns, confirmed both phenotypically and genotypically. AMR genes detected in all six isolates included *gyrA*, *gyrB*, *folA*, *dfr*, *folP*, *tet35*, the *CatB* family, *S10p*, *alr*, *murA*, *EF-G*, *H-NS*, *S12p*, *gigB*, *mphA*, *oxyR*, *kasA*, *katG*, and *EF-Tu*. Genes present in 5 isolates (83.33%) included the *SHV* family and *mox-3*, while *APH(3″)-Ib* and *APH(6”)-Id* were found in 4 isolates (66.67%). Less frequently detected genes (16.67%) included *parC*, *parE*, *qnr*, *dfrA1*, *tetA*, *tetR*, *fosA5*, *floR*, and the *CphA* family. In comparison, a study using ONT MinION sequencing of strains from Ghana reported *APH(3″)-Ib* and *APH(6″)-Id* in 74% of isolates, *catB9* and *dfrA1* in 79%, *floR* in 74%, *rsmA* in 82%, and *parC* and *parE* in 35% of isolates [41].

In this study, mutations were identified in the DNA gyrase subunit A (GyrA) at positions S83I, S177A, and S202A, and in the topoisomerase IV subunit (ParC) at positions S85L and I231V. Both enzymes are essential for DNA replication and are primary targets of quinolone and fluoroquinolone antibiotics. Mutations in these genes induce conformational changes that reduce antibiotic binding, thereby conferring resistance. Phenotypic AST confirmed resistance to quinolones and fluoroquinolones. Previously, mutations at GyrA S83I and ParC S85L have been reported in quinolone-resistant *V. cholerae* isolates from India, England, and China [42,43,44]. In our study, the mutations at GyrA positions S177A and S202A, and ParC position I231V, may be novel, but their role in resistance requires validation through functional laboratory work, molecular docking and simulation. Mutations in both GyrA and ParC are known to dramatically increase the minimum inhibitory concentration (MIC) values for fluoroquinolones [43].

To explore the evolutionary relationships and genetic relatedness of our isolates with global *V. cholerae* strains, including O1 7PET, a whole-genome SNP-based phylogenetic tree was constructed using sequences from human isolates collected worldwide between 2020 and 2024. The phylogeny demonstrates that our isolates are closely linked to regionally circulating strains, suggesting local persistence, while some strains also show evidence of global dissemination. Although all isolates cluster within the same clade, strains 666.7609, 666.7614, and 666.7618 appear regionally persistent across China, India, and Bangladesh (2021–2023), likely reflecting localised evolutionary pressure, environmental adaptation, and cross-border movement. Other isolates—666.7612, 666.7613, and 666.7615—show 100% genomic similarity with a Pakistani strain from 2022 and with strains reported from the USA (2020), Australia (2022), Lebanon (2022), France (2024), Congo (2022), and China (2021). These isolates illustrate both regional persistence and unexpected genetic connectivity across geographically distant locations, likely mediated by human travel and trade. The placement of our isolates highlights their potential role in bridging regional and global transmission networks of *V. cholerae*.

## 5. Conclusions

The 2023 cholera outbreak in Khyber Pakhtunkhwa was primarily driven by MDR *V. cholerae* Ogawa strains harbouring multiple AMR genes and mutations linked to quinolone resistance. These results underscore the critical need for enhanced genomic surveillance and stringent antibiotic stewardship to curb the spread of resistant *V. cholerae* strains in Pakistan. Furthermore, whole-genome phylogenetic analysis indicates that these isolates not only persist regionally within Asia, but also show evidence of dissemination to geographically distant countries, including Australia and the USA.

## Data Availability

Raw sequence data is available from DDBJ/ENA/GenBank databases, with the accession numbers: JBRBRX000000000 (VC01), JBRBRW000000000 (VC02), JBRBRV000000000 (VC03), JBRBRU000000000 (VC07), JBRREZ000000000 (VC09; version described here: JBRREZ010000000), and JBRBRT000000000 (VC12). The corresponding BioProject and Bio Sample identifiers are PRJNA1330148 and SAMN51438020–SAMN51438025, respectively.

## References

[1] Chowdhury F., Ross A.G., Islam M.T., McMillan N.A., Qadri F. (2022). Diagnosis, management, and future control of cholera. Clin. Microbiol. Rev..

[2] Ali M., Lopez A.L., You Y.A., Kim Y.E., Sah B., Maskery B., Clemens J. (2012). The global burden of cholera. Bull. World Health Organ..

[3] Ali M., Nelson A.R., Lopez A.L., Sack D.A. (2015). Updated global burden of cholera in endemic countries. PLoS Negl. Trop. Dis..

[4] Das R., Sobi R.A., Sultana A.-A., Nahar B., Bardhan P.K., Luke L., Fontaine O., Ahmed T. (2022). A double-blind clinical trial to compare the efficacy and safety of a multiple amino acid-based ORS with the standard WHO-ORS in the management of non-cholera acute watery diarrhea in infants and young children:“VS002A” trial protocol. Trials.

[5] Harris J.F., Ryan E.T., Calderwood S.B. (2012). Cholera. Lancet.

[6] Bagcchi S. (2022). Malawi takes on cholera outbreak amid cyclone devastation. Lancet Microbe.

[7] Nelson E.J., Harris J.B., Glenn Morris J., Calderwood S.B., Camilli A. (2009). Cholera transmission: The host, pathogen and bacteriophage dynamic. Nat. Rev. Microbiol..

[8] Grant T.-A., Balasubramanian D., Almagro-Moreno S. (2021). JMM Profile: Vibrio cholerae: An opportunist of human crises. J. Med. Microbiol..

[9] Ramamurthy T., Mutreja A., Weill F.-X., Das B., Ghosh A., Nair G.B. (2019). Revisiting the global epidemiology of cholera in conjunction with the genomics of Vibrio cholerae. Front. Public Health.

[10] Reidl J., Klose K.E. (2002). Vibrio cholerae and cholera: Out of the water and into the host. FEMS Microbiol. Rev..

[11] Ramamurthy T., Garg S., Sharma R., Bhattacharya S., Nair G.B., Shimada T., Takeda T., Karasawa T., Kurazano H., Pal A. (1993). Emergence of novel strain of Vibrio cholerae with epidemic potential in southern and eastern India. Lancet.

[12] Mutreja A., Kim D.W., Thomson N.R., Connor T.R., Lee J.H., Kariuki S., Croucher N.J., Choi S.Y., Harris S.R., Lebens M. (2011). Evidence for several waves of global transmission in the seventh cholera pandemic. Nature.

[13] Longini I.M., Yunus M., Zaman K., Siddique A., Sack R.B., Nizam A. (2002). Epidemic and endemic cholera trends over a 33-year period in Bangladesh. J. Infect. Dis..

[14] Fisher-Hoch S., Khan A., Khan M.A., Mintz E. (1993). Vibrio cholerae 0139 in Karachi, Pakistan. Lancet.

[15] Shah M.A., Mutreja A., Thomson N., Baker S., Parkhill J., Dougan G., Bokhari H., Wren B.W. (2014). Genomic epidemiology of Vibrio cholerae O1 associated with floods, Pakistan, 2010. Emerg. Infect. Dis..

[16] Sim E.M., Martinez E., Blackwell G.A., Pham D., Millan G., Graham R.M.A., Dhakal R., Wang Q., Suliman B., Jennison A.V. (2023). Genomes of Vibrio cholerae O1 Serotype Ogawa Associated with Current Cholera Activity in Pakistan. Microbiol. Resour. Announc..

[17] Chaguza C., Chibwe I., Chaima D., Musicha P., Ndeketa L., Kasambara W., Mhango C., Mseka U.L., Bitilinyu-Bangoh J., Mvula B. (2024). Genomic insights into the 2022–2023 Vibrio cholerae outbreak in Malawi. Nat. Commun..

[18] Baddam R., Sarker N., Ahmed D., Mazumder R., Abdullah A., Morshed R., Hussain A., Begum S., Shahrin L., Khan A.I. (2020). Genome Dynamics of Vibrio cholerae Isolates Linked to Seasonal Outbreaks of Cholera in Dhaka, Bangladesh. mBio.

[19] Lassalle F., Al-Shalali S., Al-Hakimi M., Njamkepo E., Bashir I.M., Dorman M.J., Rauzier J., Blackwell G.A., Taylor-Brown A., Beale M.A. (2023). Genomic epidemiology reveals multidrug-resistant plasmid spread between Vibrio cholerae lineages in Yemen. Nat. Microbiol..

[20] Bilal H., Khan M.N., Rehman T., Hameed M.F., Yang X. (2021). Antibiotic resistance in Pakistan: A systematic review of past decade. BMC Infect. Dis..

[21] Din J.U., Hassan M., Ihsan N., Amin A., Ali I., Khan A., Mumtaz A. (2022). Socio-Economic Implications of Terrorism on Khyber Pakhtunkhwa: A Case Study of ANP Era (2008–2013). Webology.

[22] Shrestha B.K., Tumbahangphe M., Shakya J., Chauhan S., Dhungana B., Shrestha R., Limbu J., Khadka K., Rai A. (2020). Laboratory protocols for isolation and identification of toxigenic strains of Vibrio cholerae: A review. Int. J. Res. Appl. Sci. Biotechnol. (IJRASB).

[23] Cheesbrough M. (2005). District Laboratory Practice in Tropical Countries, Part 2.

[24] Thaotumpitak V., Sripradite J., Atwill E.R., Jeamsripong S.J.P. (2023). Emergence of colistin resistance and characterization of antimicrobial resistance and virulence factors of *Aeromonas hydrophila*, *Salmonella* spp., and *Vibrio cholerae* isolated from hybrid red tilapia cage culture. PeerJ.

[25] Smith P., Le Devendec L., Jouy E., Larvor E., Lesne J., Kirschner A.K., Rehm C., Leopold M., Pleininger S., Heger F.J. (2023). Epidemiological cut-off values for non-O1/non-O139 Vibrio cholerae disc diffusion data generated by standardised methods. Dis. Aquat. Org..

[26] Karatuna O., Matuschek E., Åhman J., Caidi H., Kahlmeter G. (2024). *Vibrio* species: Development of EUCAST susceptibility testing methods and MIC and zone diameter distributions on which to determine clinical breakpoints. J. Antimicrob. Chemother..

[27] Hindler J., Richter S., Bernard K., Jones S., Castanheira M., Citron D., Couturier M., Fritsche T., Humphries R., Jorgensen J. (2016). CLSI M45-Methods for an-Timicrobial Dilution and Disk Susceptibility Testing of Infrequently Isolated or Fastidious Bacteria.

[28] Basak S., Singh P., Rajurkar M. (2016). Multidrug resistant and extensively drug resistant bacteria: A study. J. Pathog..

[29] Nayak D.S.K., Das J., Swarnkar T. (2022). Quality control pipeline for next generation sequencing data analysis. Intelligent and Cloud Computing: Proceedings of ICICC 2021.

[30] Bankevich A., Nurk S., Antipov D., Gurevich A.A., Dvorkin M., Kulikov A.S., Lesin V.M., Nikolenko S.I., Pham S., Prjibelski A.D. (2012). SPAdes: A new genome assembly algorithm and its applications to single-cell sequencing. J. Comput. Biol..

[31] Chklovski A., Parks D.H., Woodcroft B.J., Tyson G.W. (2023). CheckM2: A rapid, scalable and accurate tool for assessing microbial genome quality using machine learning. Nat. Methods.

[32] Seemann T. (2014). Prokka: Rapid prokaryotic genome annotation. Bioinformatics.

[33] Wattam A.R., Davis J.J., Assaf R., Boisvert S., Brettin T., Bun C., Conrad N., Dietrich E.M., Disz T., Gabbard J.L. (2017). Improvements to PATRIC, the all-bacterial bioinformatics database and analysis resource center. Nucleic Acids Res..

[34] Letunic I., Bork P. (2021). Interactive Tree Of Life (iTOL) v5: An online tool for phylogenetic tree display and annotation. Nucleic Acids Res..

[35] Mashe T., Domman D., Tarupiwa A., Manangazira P., Phiri I., Masunda K., Chonzi P., Njamkepo E., Ramudzulu M., Mtapuri-Zinyowera S.J. (2020). Highly resistant cholera outbreak strain in Zimbabwe. N. Engl. J. Med..

[36] Bitew A., Gelaw A., Wondimeneh Y., Ayenew Z., Getie M., Tafere W., Gebre-Eyesus T., Yimer M., Beyene G.T., Bitew M. (2024). Prevalence and antimicrobial susceptibility pattern of Vibrio cholerae isolates from cholera outbreak sites in Ethiopia. BMC Public Health.

[37] Awuor S.O., Omwenga E.O., Daud I.I. (2020). Geographical distribution and antibiotics susceptibility patterns of toxigenic Vibrio cholerae isolates from Kisumu County, Kenya. Afr. J. Prim. Health Care Fam. Med..

[38] Chatterjee P., Kanungo S., Bhattacharya S.K., Dutta S. (2020). Mapping cholera outbreaks and antibiotic resistant Vibrio cholerae in India: An assessment of existing data and a scoping review of the literature. Vaccine.

[39] Garbern S.C., Chu T.-C., Yang P., Gainey M., Nasrin S., Kanekar S., Qu K., Nelson E.J., Leung D.T., Ahmed D. (2021). Clinical and socio-environmental determinants of multidrug-resistant Vibrio cholerae 01 in older children and adults in Bangladesh. Int. J. Infect. Dis..

[40] De R. (2021). Mobile genetic elements of Vibrio cholerae and the evolution of its antimicrobial resistance. Front. Trop. Dis..

[41] Fuesslin V., Krautwurst S., Srivastava A., Winter D., Liedigk B., Thye T., Herrera-León S., Wohl S., May J., Fobil J.N. (2022). Prediction of Antibiotic Susceptibility Profiles of Vibrio cholerae Isolates From Whole Genome Illumina and Nanopore Sequencing Data: CholerAegon. Front. Microbiol..

[42] Zhou Y.-Y., Ma L.-Y., Yu L., Lu X., Liang W.-L., Kan B., Su J.-R. (2023). Quinolone resistance genes and their contribution to resistance in Vibrio cholerae serogroup O139. Antibiotics.

[43] Samal D., Turuk J., Nayak S.R., Pany S., Pal B.B., Pati S. (2025). Genomic insights into the dynamic antibiotic resistance landscape of Vibrio cholerae during the Cholera outbreak 2022 in Odisha, India. Sci. Rep..

[44] Nair S., Barker C.R., Patel V., Poh C.-Y., Greig D.R., Olonade I., Ribeca P., Jenkins C. (2025). Highly drug-resistant Vibrio cholerae harbouring bla PER-7 isolated from travellers returning to England. J. Antimicrob. Chemother..

